# Bi-Layer Shortest-Path Network Interdiction Game for Internet of Things

**DOI:** 10.3390/s20205943

**Published:** 2020-10-21

**Authors:** Jingwen Yan, Kaiming Xiao, Cheng Zhu, Jun Wu, Guoli Yang, Weiming Zhang

**Affiliations:** 1 Science and Technology on Information Systems Engineering Laboratory, National University of Defense Technology, Changsha 410073, China; kmxiao@nudt.edu.cn (K.X.); zhucheng@nudt.edu.cn (C.Z.); yangguoli@nudt.edu.cn (G.Y.); wmzhang@nudt.edu.cn (W.Z.); 2 International Academic Center of Complex Systems, Beijing Normal University, Zhuhai 519087, China; junwu@nudt.edu.cn; 3 Rural Vitalization Research Institute, Changsha University, Changsha 410073, China

**Keywords:** network interdiction, Internet of Things, logical–physical networks, shortest path, Benders decomposition

## Abstract

Network security is a crucial challenge facing Internet-of-Things (IoT) systems worldwide, which leads to serious safety alarms and great economic loss. This paper studies the problem of malicious interdicting network exploitation of IoT systems that are modeled as a bi-layer logical–physical network. In this problem, a virtual attack takes place at the logical layer (the layer of Things), while the physical layer (the layer of Internet) provides concrete support for the attack. In the interdiction problem, the attacker attempts to access a target node on the logical layer with minimal communication cost, but the defender can strategically interdict some key edges on the physical layer given a certain budget of interdiction resources. This setting generalizes the classic single-layer shortest-path network interdiction problem, but brings in nonlinear objective functions, which are notoriously challenging to optimize. We reformulate the model and apply Benders decomposition process to solve this problem. A layer-mapping module is introduced to improve the decomposition algorithm and a random-search process is proposed to accelerate the convergence. Extensive numerical experiments demonstrate the computational efficiency of our methods.

## 1. Introduction

With the development of information and communication technologies, more and more functional systems have begun to be built based on information networks. Thanks to the high-speed and massive data transmission of the information network, the scale and efficiency of the functional system has been greatly increased. It makes the multi-layer network system represented by Internet of Things (IoT) widely used in various fields such as energy, health care, communication, transportation and manufacturing [1]. The combination of networks of different layers makes the whole system have far more powerful and intelligent functions than ever before. However, the high integration and connectivity of IoT make them more vulnerable to malicious attacks [2,3,4]. An error or failure of a certain layer may have serious effects on the entire system, and the malicious attacks on the system may be carried out from multiple layers, e.g., the cascading failures of the Italian smart grid on 28 September 2003 [5] and the Stuxnet worm against Iranian nuclear facilities [6]. The great significance and vulnerability of IoT has inspired researchers to pay attention to the security protection of them. Recent literature has carried out relevant research on the security of IoT and other layered network systems from different perspectives [7,8,9,10,11,12,13,14].

**Figure 1:**
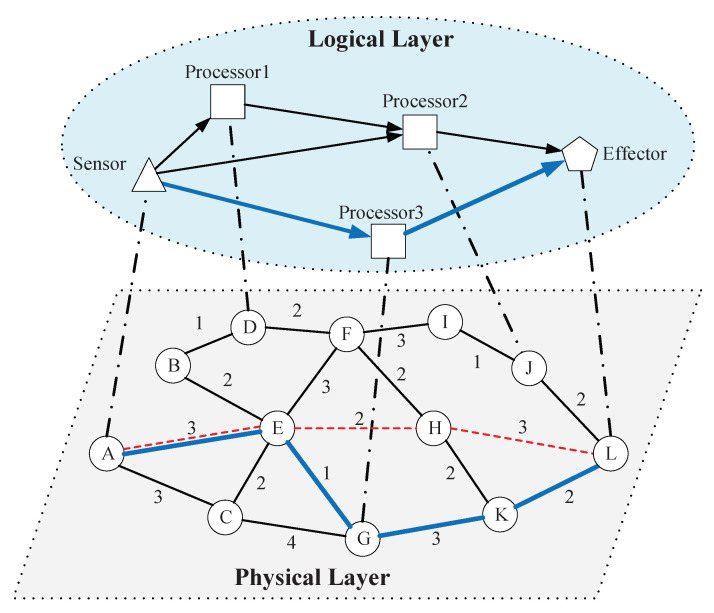
A simple example of the logical–physical network in IoT. The logical layer contains a sensor, an effector and three different processors. The physical layer is the communication network, where the time delay of each link is given by the number beside it. Each dotted line between the two layers connects the functional part and the communicating part of the same entity. The sensor collects information and sends it to either of the processors. The processor analyzes the information and then sends order to the effector. As the figure shows, the shortest logical flow *Sensor → Processor3 → Effector* corresponds to a physical path *A(Sensor) → E → G(Processor 3) → K → L(Effector)*, which weighs 9. Although the shortest *A*–*L* path on the physical layer is *A(Sensor) → E → H → L(Effector)* with total weight 8, it is not functionally feasible because no processor is on this path and thus no effective order can be sent to Effector.

On some occasions, we need to interdict the layered network of functional systems to defend against malicious attacks. For example, criminals invade the IoT and occupy some important devices, attempting to issue malicious instructions to destroy key facilities. The network operators hope to delay the criminals’ attack by interdicting some links to buy time for the deployment of heavyweight countermeasures [9]. Such malicious attacks usually spread through direct connections in the network, expand the scope of influence, and ultimately achieve functional damage. To characterize the propagation behavior of malicious attacks in layered network systems such as IoT and to study the defense strategy, a suitable network model is necessary. The logical–physical network model (Concept of logical–physical network. Retrieved from https://www.ibm.com/support/knowledgecenter/en/SSPHQG_7.2/concept/ha_concepts_physical_logical.html), which provides us a proper approach to modeling IoT and other real layered network systems, is used in this paper. IoT is a huge network which combines functional devices with the Internet [15]. In IoT, there are functional collaboration and dependency relationships among smart devices such as information-sensing devices, information-processing devices, information-fusion devices, and effectors. In the framework of logical–physical network models, the network formed by these devices and relationships describes how the system performs tasks and achieves functions, and we call it the logical-layer network. Before being functionally connected, these devices need to be physically connected through the Internet, i.e., they need to transfer information through the actual communication network, which we call the physical-layer network. The specific realization of system functions, on the logical layer, needs devices to cooperate according to certain organizational rules; on the physical layer, needs devices to transmit information through actual communication pathways. Figure 1 shows how the functional processes in the logical-layer network correspond to real physical communication paths, and the difference from the single-layer case.

A specific system function, which is determined by a starting node $s_{l}$ and an ending node $t_{l}$, may have different implementations on the logical layer, and these logical implementations may correspond to different communication paths. In the sense of optimization, the communication paths are designed to be shortest (i.e., has minimum delay).

How to deploy an effective interdiction strategy when the logical–physical network suffers malicious intrusion is worthy of attention. Appropriate interdiction strategies can earn sufficient time for network operators, and the optimization of interdiction strategies, which we call the logical–physical network shortest-path interdiction (LPNSPI) problem, is exactly what we want to solve in this paper. LPNSPI is a zero-sum game involving two players: an attacker and a defender. The attacker’s goal is to find a path which has minimum delay between a particular origin–destination pair in the logical-layer network, and the defender aims to delay the path as much as possible by interdicting connections in the physical-layer network. LPNSPI is an expansion and supplement of the shortest-path network interdiction (SPNI), which is a derivative of the network interdiction problem (NIP), on logical–physical networks. As an important issue in network attack and defense, NIP has applications in various fields, such as the supply-chain network [16], infrastructure protection [17], interdicting nuclear proliferation [18], etc.

NIP is also a useful model for researching the protection of IoT, e.g., the defensive resource allocation [15] and adversarial outbreak detection [19]. Numerous variations of NIP have been proposed and studied to meet different hypotheses under particular scenarios [16,17,20,21,22,23,24]. Among the many types of NIP, SPNI is a classic and important branch. Generally, SPNI can be described as *maximizing the shortest path* and may be formulated as a bi-level mixed-integer program. Researchers have applied the SPNI model to transportation [25,26], port security, border patrol and other aspects [27]. However, most of the literature about SPNI focuses on the single-layer network where interdiction and pathfinding happen in the same layer [28,29]. In LPNSPI, interdiction is applied on the physical layer, but the path that the attacker wants to minimize is on the logical layer. With the physical-layer network alone, it is impossible to judge whether a path meets the functional requirements; with the logical-layer network alone, it is impossible to confirm the specific delay of links in the network and the effect of interdiction. The separation of these two layers brings about the non-linearity of the objective function (In Section 2, we present the non-linearity in LPNSPI. The two-layer structure leads to the product term of decision variables in the objective function (Equation (7))), and also make the traditional single-layer NIP solving methods unable to be directly applied to LPNSPI. Recently the layered network interdiction problem has attracted more attention. Kennedy [30] studied the maximum flow interdiction problem in a kind of multi-layer network where different layers are connected by sharing some common elements (nodes or edges). Wei et al. [31] studied the shortest-path interdiction problem in a kind of bi-layer network where the interdiction effects on one layer can be determined by the interdiction on the other layer through logical operations. Baycik et al. [32] studied the interdiction problem in layered physical and information flow networks. In this problem, a physical node can only be used when the flow passing through its corresponding information-layer node is more than a particular demand. Compared to this research, LPNSPI has a completely different setting on the inter-layer relationships of logical–physical networks, which pays more attention to the relationships between logical functions and physical communication paths. Table 1 briefly compares some of the characteristics of LPNSPI and layered network interdiction problems mentioned above.

In this paper, we propose the LPNSPI game and model it as a bi-level integer program. We reformulate the problem and develop a Benders decomposition algorithm framework to solve LPNSPI. There are two major approaches to solve SPNI problems: the decomposition method and the dual method. The advantages of the decomposition approach have been mentioned in [33], and as shown in Table 1, the dual method is not suitable for LPNSPI. Then we propose a Layer-Mapping module to reduce unnecessary calculations of the decomposition algorithm. Also, a Random-Search module is developed to accelerate the convergence of the algorithm with a given approximate ratio. Simulation experiments are designed, and the computational results prove the significant efficiency of Layer-Mapping and Random-Search. Finally, we test our algorithms in a real bi-layer IoT network, and our improving methods perform well in both solving time and interdiction effects.

The paper is organized as follows. In Section 2, the LPNSPI model is defined and formulated. In Section 3, a basic decomposition algorithm is developed after reformulation. Layer-Mapping is introduced in Section 4, and Random-Search is proposed in Section 5. Section 6 provides the experimental results. Conclusions are in Section 7.

## 2. Shortest Path Interdiction Problem in Logical–Physical Networks

The LPNSPI problem involves two different networks: the logical-layer network which represents the flow of information between logical entities, and the physical-layer network which represents the actual transmission path of information in a physical environment. Throughout the present work, we use normal symbols to represent scalars, and bold symbols for vectors/matrices. The logical-layer network is defined as a directed graph $G_{l}=\left(N_{l},A_{l}\right)$, where $N_{l}$ represents the set of logical nodes and $A_{l}$ represents the set of logical arcs. Each logical node corresponds to a logical entity such as a person, a unit, an organization, etc. Each logical arc $e_{l}=\left(i_{l},j_{l}\right)$ represents an allowed information transfer direction between logical nodes. The direction of a logical link is generally defined by artificial rules (such as process rules, hierarchy rules, etc.) rather than natural ones. For instance, in the logical network, two nodes are adjacent because they may have a direct functional dependency (the proper functioning of node *i* depends on the processed information provided by node *j*) rather than because they have a direct physical connection. Unlike a logical-layer network, a physical-layer network is defined as a bidirected graph $G_{p}=\left(N_{p},A_{p}\right)$, where $N_{p}$ represents the set of physical nodes and $A_{p}$ represents the set of physical arcs. Physical arcs are bidirected because they correspond to connections in the actual physical environment, such as road connections, routing connection, and so on. These connections are not directional in themselves and the flow of information on them can be two-way. Each logical node $i_{l}$ has a corresponding physical node $i_{p}$, but not necessarily the other way around (many nodes in the physical network serve only as information transfer nodes and are not necessary to perform system functions). Each physical arc $e_{p}=\left(i_{p},j_{p}\right)\in A_{p}$ has a cost of communication $c_{e_{p}}$ for the attacker, which will be increased to $c_{e_{p}}+d_{e_{p}}$ if the arc is interdicted by the defender. In addition, for the defender, the corresponding resource consumption of interdicting $e_{p}$ is denoted as $r_{e_{p}}$. The total interdiction resource for the defender is *R*. The communication cost of a logical arc $w_{e_{l}}$ is the total communication cost of a path that the attacker choose to travel in the physical-layer network, the corresponding node of endpoints of the logical arc being the start node and end node of the physical path.

In this problem, we assume both the attacker and the defender have complete information about the network. The defender pre-deploys the defense strategy according to the attacker’s source node $s_{l}$ and target node $t_{l}$, and blocks some edges in the network. Subsequently, the attacker develops an optimal attack plan to minimize the communication cost from the starting node to the target node. Let $x_{e_{p}}$ (the vector form is denoted by bold x) denote the defender’s interdiction strategy on $e_{p}$, and let $y_{e_{l}}$ (the vector form is denoted by bold y) denote the attacker’s pathfinding variable on the logical layer. Then the attacker’s problem can be formulated as follows:

(1) $$\begin{array}{ccc} & \min_{y} & \sum_{e_{l}\in A_{l}}w_{e_{l}}y_{e_{l}} \end{array}$$

(2) $$\begin{array}{ccc} & s.t. & \sum_{e_{l}\in FS\left(v_{l}\right)}y_{e_{l}}-\sum_{e_{l}\in RS\left(v_{l}\right)}y_{e_{l}}=\left\{\begin{array}{cc} 1 & if\;v_{l}=s_{l}\\ -1 & if\;v_{l}=t_{l}\\ 0 & else \end{array}\;\;,\forall v_{l}\in N_{l}\right. \end{array}$$

(3) $$\begin{array}{ccc} & & y_{e_{l}}\in \left\{0,1\right\},\;\forall e_{l}\in A_{l} \end{array}$$

where $w_{e_{l}}$ is the weight of the logical link $e_{l}=\left(i_{l},j_{l}\right)$, i.e., the minimum total cost of its corresponding paths on the physical layer:

(4) $$\begin{array}{ccc} w_{e_{l}}=w_{\left(i_{l},j_{l}\right)}= & \min_{k} & \sum_{e_{p}\in A_{p}}\left(c_{e_{p}}+x_{e_{p}}d_{e_{p}}\right)k_{e_{l}e_{p}} \end{array}$$

(5) $$\begin{array}{ccc} & s.t. & \sum_{e_{p}\in FS\left(v_{p}\right)}k_{e_{l}e_{p}}-\sum_{e_{p}\in RS\left(v_{p}\right)}k_{e_{l}e_{p}}=\left\{\begin{array}{ccc} 1 & & if\;v_{p}=i_{p}\\ -1 & & if\;v_{p}=j_{p}\\ 0 & & else \end{array},\;\;\forall v_{p}\in N_{p}\right. \end{array}$$

(6) $$\begin{array}{ccc} & & k_{e_{l}e_{p}}\in \left\{0,1\right\},\;\;\forall e_{p}\in A_{p} \end{array}$$

where $k_{e_{l}e_{p}}$ (Let K denotes the matrix form) is the pathfinding variable which indicates whether $e_{p}$ is chosen in the corresponding physical path of $e_{l}$. $x_{e_{p}}=1$ when the physical arc $e_{p}$ is interdicted and $x_{e_{p}}=0$ otherwise. $k_{e_{l}e_{p}}=1$ indicates that $e_{p}$ is in the physical shortest path corresponding to $e_{l}$. $FS(v_{p})$ and $RS(v_{p})$ represent respectively the arc set directed out of and into node $v_{p}$. $i_{p}$ and $j_{p}$ are respectively the corresponding physical nodes of $i_{l}$ and $j_{l}$. Constraint (2) and (5) are the flow-balance constraints. In practice, $w_{e_{l}}$ can be calculated by using common shortest-path algorithms such as the Dijkstra algorithm.

We define $V_{s_{l}t_{l}}$ as the total communication cost from $s_{l}$ to $t_{l}$. Then the defender’s problem of maximizing $V_{s_{l}t_{l}}$, which is exactly the LPNSPI problem, can be formulated as follows

$$\begin{array}{ccc} \left[\mathrm{LPNSPI}\right] & V_{s_{l}t_{l}}^{*} & =\max_{x}\;\min_{y}V_{s_{l}t_{l}}=\max_{x}\;\min_{y}\sum_{e_{l}\in A_{l}}w_{e_{l}}\left(x\right)y_{e_{l}} \end{array}$$

(7) $$\begin{array}{ccc} & & =\max_{x}\;\min_{y,k}\sum_{e_{l}\in A_{l}}\left[\sum_{e_{p}\in A_{p}}\left(c_{e_{p}}+x_{e_{p}}d_{e_{p}}\right)k_{e_{l}e_{p}}\right]y_{e_{l}} \end{array}$$

(8) $$\begin{array}{ccc} & s.t. & \sum_{e_{l}\in FS\left(v_{l}\right)}y_{e_{l}}-\sum_{e_{l}\in RS\left(v_{l}\right)}y_{e_{l}}=\left\{\begin{array}{cc} 1 & if\;v_{l}=s_{l}\\ -1 & if\;v_{l}=t_{l}\\ 0 & else \end{array}\;\;,\forall v_{l}\in N_{l}\right. \end{array}$$

(9) $$\begin{array}{ccc} & & \sum_{e_{p}\in FS\left(v_{p}\right)}k_{e_{l}e_{p}}-\sum_{e_{p}\in RS\left(v_{p}\right)}k_{e_{l}e_{p}}=\left\{\begin{array}{ccc} & 1 & if\;v_{p}=i_{p}\\ & -1 & if\;v_{p}=j_{p}\\ & 0 & else \end{array},\;\;\forall v_{p}\in N_{p}\right.,\forall e_{l}=\left(i_{l},j_{l}\right)\in A_{l} \end{array}$$

(10) $$\begin{array}{ccc} & & x_{e_{p}}=x_{\overset{\leftarrow }{e_{p}}},\;\forall e_{p}\in A_{p} \end{array}$$

(11) $$\begin{array}{ccc} & & \sum_{e_{p}\in A_{p}}x_{e_{p}}r_{e_{p}}\leq 2R \end{array}$$

(12) $$\begin{array}{ccc} & & x_{e_{p}},y_{e_{l}},k_{e_{l}e_{p}}\in \left\{0,1\right\},\;\;\forall e_{p}\in A_{p},\;\forall e_{l}\in A_{l} \end{array}$$

where $\overset{\leftarrow }{e_{p}}$ is the reverse arc of $e_{p}$ and constraint (10) indicates that the interdiction of an arc is effective for both directions. Constraint (11) is the resource constraint for the defender, where we use 2R as the resource limit because of the counting for both directions. It is noted that the objective function (7) is nonlinear, which results from the mapping relationship between the physical-layer network and the logical-layer network. A Benders decomposition algorithm framework and related improvement methods are proposed in this paper.

## 3. Basic Decomposition Algorithm for LPNSPI

The problem of shortest-path network interdiction can be naturally divided into two processes: blocking resource deployment process and pathfinding process. These two processes respectively correspond to the max operation and min operation in (7), and correspond to the master problem and subproblem of the Benders decomposition algorithm. Let d denote the vector of $d_{e_{p}}$ and D=diag(d). $\hat{z}$ denotes an $s_{p}$–$t_{p}$ path on the physical layer and $\hat{Z}$ denotes a collection of $s_{p}$–$t_{p}$ paths. The master problem and the subproblem of LPNSPI are defined as follows:

(13) $$\begin{array}{ccc} {}[\mathrm{Master}(\hat{Z})] & & V_{\hat{Z}}=\max_{x}V \end{array}$$

(14) $$\begin{array}{ccc} & s.t. & V\leq c^{T}\hat{z}+x^{T}D\hat{z},\;\;\forall \hat{z}\in \hat{Z}\\ & & \mathrm{Constraint}\;\left(10\right)\;\mathrm{and}\;\mathrm{Constraint}\;\left(11\right) \end{array}$$

(15) $$\begin{array}{ccc} & & x_{e_{p}}\in \left\{0,1\right\},\;\;\forall e_{p}\in A_{p} \end{array}$$

(16) $$\begin{array}{ccc} {}[\mathrm{Sub}(\hat{x})] & & V_{\hat{x}}=\min_{z}\sum_{e_{p}\in A_{p}}\left(c_{e_{p}}+{\hat{x}}_{e_{p}}d_{e_{p}}\right)z_{e_{p}}\\ & s.t. & \mathrm{Constraint}\;\left(8\right)\;\mathrm{and}\;\mathrm{Constraint}\;\left(9\right) \end{array}$$

(17) $$\begin{array}{ccc} & & z_{e_{p}}=\sum_{e_{l}\in A_{l}}y_{e_{l}}k_{e_{l}e_{p}},\;\;\forall e_{l}\in A_{l} \end{array}$$

(18) $$\begin{array}{ccc} & & y_{e_{l}},k_{e_{l}e_{p}}\in \left\{0,1\right\},\;\;\forall e_{p}\in A_{p},\;\forall e_{l}\in A_{l} \end{array}$$

In contrast to the case in a single-layer network, $\hat{z}$ does not necessarily represent a simple path. For the attacker, searching a path with minimum communication cost will lead to the shortest path in the logical layer, which is definitely a simple path. Although the corresponding path in physical and may have repeated arcs, the attacker cannot avoid going through them because the topology of logical layer specifies the process that the attacker must follow to achieve his goal.

Let *Z* denote the set of shortest physical layer paths that all simple $s_{l}$-$t_{l}$ paths in logical layer correspond. Notice that $\hat{z}\in Z$ is always established, then [Master($\hat{Z}$)] is an equivalent formulation of [LPNSPI] when $\hat{Z}=Z$. Benders decomposition algorithm fixes $\hat{Z}$ and $\hat{x}$ in turn, and iteratively solves the master problem and subproblem in turn. [Master($\hat{Z}$)] fixes the set of feasible paths $\hat{Z}$ and solves an optimal interdiction strategy from the aspect of the defender, while [Sub($\hat{x}$)] gives an optimal path selection with fixed logical-layer network status, standing at the angle of the attacker.

Israeli and Wood [33] proposed two types of “supervalid inequalities” (SVI) constraints to strengthen the LP relation of the master problem of Benders decomposition for the shortest-path interdiction problem of single-layer networks. These inequalities are constructed after the subproblem gives a currently optimal path and added to the master problem as constraints. SVIs are based on the following idea: they may make some solutions infeasible but are guaranteed not to eliminate any optimal solutions unless the incumbent is itself optimal; by reducing the size of the feasible region, SVIs accelerate the master problem. These inequalities can be extended to the logical–physical networks, and the proofs are basically the same with the single-layer case. The master problem containing SVIs is as follows:

(19) $$\begin{array}{ccc} {}[\mathrm{Master}(\hat{Z})-\mathrm{SVI}] & & V_{\hat{Z}}=\max_{x}V \end{array}$$

(20) $$\begin{array}{ccc} s.t. & & V\leq c^{T}\hat{z}+x^{T}D\hat{z},\;\;\forall \hat{z}\in \hat{Z} \end{array}$$

(21) $$\begin{array}{ccc} & & {\hat{z}}^{T}x\geq p+1,\;\;\forall \hat{z}\in \hat{Z} \end{array}$$

(22) $$\begin{array}{ccc} & & {\tilde{z}}^{T}x\geq 1,\;\;\forall \hat{z}\in \hat{Z}\\ & & \mathrm{Constraint}\;\left(10\right)\;\mathrm{and}\;\mathrm{Constraint}\;\left(11\right) \end{array}$$

(23) $$\begin{array}{ccc} & & x_{e_{p}}\in \left\{0,1\right\},\;\;\forall e_{p}\in A_{p} \end{array}$$

where $c^{T}\hat{z}+\sum_{h=1}^{p+1}d_{m_{h}}{\hat{z}}_{m_{h}}>\underline{V}\geq c^{T}\hat{z}+\sum_{h=1}^{p}d_{m_{h}}{\hat{z}}_{m_{h}}$ for p≤H and the sequence $\left\{d_{m_{h}}{\hat{z}}_{m_{h}}\right\}$. $\left\{d_{m_{h}}{\hat{z}}_{m_{h}}\right\}$ is the descending order of the sequence $\left\{d_{h}{\hat{z}}_{h}\right\}$ (the number of terms is *H*). $\tilde{z}=\left(diag\left(1-\hat{x}\right)\right)\hat{z}$. Constraint (21) is the Type-I SVI and Constraint (22) is the Type-II SVI. For detailed introduction and related proofs of SVI, please refer to [33].

Because of the logical–physical structure, [Sub($\hat{x}$)] contains nonlinear terms and cannot be solved directly. However, we can divide the solution process into two steps: first, calculate the current communication cost $\hat{w}$ of the logical layer links with the current interdiction strategy $\hat{x}$, and then calculate the shortest path of the logical layer. Here we rewrite the subproblem as follows:

(24) $$\begin{array}{ccc} {}[\mathrm{Sub}(\hat{w})-\mathrm{LM}] & & V_{\hat{w}}=\min_{y}\sum_{e_{l}\in A_{l}}{\hat{w}}_{e_{l}}y_{e_{l}}\\ & s.t. & \mathrm{Constraint}\;\left(2\right)\;\mathrm{and}\;\mathrm{Constraint}\;\left(3\right) \end{array}$$

We denote the matrix K obtained when calculating $\hat{w}$ as $\hat{K}$, and denote the current logical path given by [Sub($\hat{w}$)-LM] as $\hat{y}$, then we have $\hat{z}={\hat{K}}^{T}\hat{y}$. [Sub($\hat{w}$)-LM] is also a necessary reformulation of the subproblem in order to use Layer-Mapping, which we will introduce in the next section. Now we give the basic decomposition algorithm for LPNSPI:
**Algorithm 1** Basic Benders decomposition algorithm for LPNSPI **Input** : An instance of LPNSPI**Output**: An optimal interdiction plan $x^{*}$ 1: $\hat{x}\leftarrow 0;\hat{Z}\leftarrow \emptyset ;\underline{V}\leftarrow -\infty ;\bar{V}\leftarrow \infty$
2: **while** 
$\bar{V}-\underline{V}>0$ 
**do**3:  Calculate $\hat{w}$ and $\hat{K}$ according to $\hat{x}$ using (4)-(6)4:  Solve [Sub($\hat{w}$)-LM] for $\hat{y}$ and the objective value $V_{\hat{w}}$
5:  $\hat{z}={\hat{K}}^{T}\hat{y}$; $\hat{Z}\leftarrow \hat{Z}\cup \hat{z}$;6:  
**if** 
$\underline{V}<V_{\hat{w}}$ 
**then**
7:   $x^{\prime }\leftarrow \hat{x}$; $\underline{V}\leftarrow V_{\hat{w}}$;
8:  **end if**9:  
**if** 
$\bar{V}-\underline{V}\leq 0$ 
**then**
10:   **break**;11:  **end if**12:  Solve [Master($\hat{Z}$)-SVI] for $\hat{x}$ and the objective value $V_{\hat{Z}}$;
13:  $\bar{V}\leftarrow V_{\hat{Z}}$;
14: **end while**15:
$x^{*}\leftarrow x^{\prime }$
16:
**return** 
$x^{*}$



The correctness of Algorithm 1 is based on the following facts: $V_{\hat{w}}$ gives a lower bound on the attacker’s optimal objective value and $V_{\hat{Z}}$ gives a upper bound on the defender’s optimal objective value. Although the actual path in the physical layer z may no longer be a simple path in the case of layered network, the corresponding path in logical layer y is certainly simple, whose number is finite. The number of possible interdiction plans x is also finite. In addition, once y and x are fixed, z is fixed, which means that the algorithm converges in a finite number of iterations.

## 4. Layer-Mapping Module

As shown in Algorithm 1, during the iteration of the decomposition algorithm, the current interdiction strategy $\hat{x}$ changes when the master problem finds a better solution. In addition, the change of the interdiction strategy results in the change of the network status which can be represented by $\hat{w}$ and $\hat{K}$: $\hat{w}$ indicates the weights of logical links and $\hat{K}$ indicates the corresponding relationships between logical links and physical paths. Once the current network status ($\hat{w}$ and $\hat{K}$) is determined, we can solve a relatively simple subproblem [Sub($\hat{w}$)-LM] ([Sub($\hat{w}$)-LM] shares the same representation form with the subproblem of the single-layer case ) and give the currently optimal logical path $\hat{y}$. However, this process (line 3 in Algorithm 1) contains a lot of double counting during the iteration because we recalculate ${\hat{w}}_{e_{l}}$ and ${\hat{k}}_{e_{l}e_{p}}$ for every logical link and physical arc in each iteration. It inspires us to add a Layer-Mapping module between the master problem and the subproblem to find the specific changes of the network status when $\hat{x}$ changes.

Since the interdiction impact $d_{e_{p}}$ is positive, interdicting a physical arc will not shorten any physical shortest path. So, the change of $\hat{x}$ has no effect on a physical shortest path (that means this path is still shortest) if no arc of it is interdicted. Let $x^{0}$ denote an initial interdiction strategy. Let $w^{0}$ denote the corresponding weights (communication costs) of logical links and let $K^{0}$ denote the corresponding path-mapping matrix K. When the interdiction strategy changes to $\hat{x}$ which satisfies $\left\{e_{p}|x_{e_{p}}^{0}=1\right\}\subseteq \left\{e_{p}|{\hat{x}}_{e_{p}}=1\right\}$, we need only to recalculate ${\hat{w}}_{e_{l}}$ and ${\hat{k}}_{e_{l}e_{p}}$ for logical link $e_{l}$ if there exists a physical arc $e_{p}\in \left\{e_{p}\in A_{p}|x_{e_{p}}^{0}\neq {\hat{x}}_{e_{p}}\right\}$ which makes $K_{e_{l}e_{p}}^{0}=1$. In practice, we set $x^{0}$ to 0, and thus $K^{0}$ indicates the initial network status when there is no interdiction. When the resource limit makes the number of physical arcs that can be interdicted simultaneously much smaller than the edge number of the physical network, layer-mapping will significantly reduce the weight calculations for logical links.
**Algorithm 2** Layer-Mapping **Input**: Initial interdiction strategy $x^{0}$; initial network status $w^{0}$ and $K^{0}$; the new interdiction strategy $\hat{x}$
**Output**: The new network status $\hat{w}$ and $\hat{K}$;
 1: Initialize $\hat{w}$ and $\hat{K}$
2:
$\hat{Diff}=\left\{e_{p}\in A_{p}|{x^{0}}_{e_{p}}\neq {\hat{x}}_{e_{p}}\right\}$
3:
**for** 
$e_{l}\in A_{l}$ 
**do**
4:  flag ← 0;
5:  
**for** 
$e_{p}\in \hat{Diff}$ 
**do**
6:    
**if** 
$K_{e_{l}e_{p}}^{0}=1$ 
**then**
7:      Calculate ${\hat{w}}_{e_{l}}$ and ${\hat{K}}_{e_{l}}$ using (4)-(6);
8:      flag ← 1;9:      **break**;
10:    **end if**11:  **end for**12:  **if** flag = 0 **then**
13:    
${\hat{w}}_{e_{l}}\leftarrow {w0}_{e_{l}},{\hat{K}}_{e_{l}}\leftarrow {K^{0}}_{e_{l}}$
14:    **end if**15: **end for**
16: **return** $\hat{w}$ and $\hat{K}$



${\hat{K}}_{e_{l}}$ in Layer-Mapping is the vector of ${\hat{k}}_{e_{l}e_{p}}$ when $e_{l}$ is fixed (it can also be explained as the $e_{l}$th row vector of matrix $\hat{K}$), and ${K^{0}}_{e_{l}}$ shares the same representation. Layer-Mapping intuitively shows the change of the mapping status of the logical–physical network when interdiction happens, which not only avoids the non-linearity of the objective function from explicitly appearing in the decomposition algorithm, but also speeds up the solution of the subproblem. For simplicity, we call this module Layer-Mapping($x^{0}$,$\hat{x}$), where $x^{0}$ is the initial interdiction strategy and $\hat{x}$ is the new one. Applying Layer-Mapping to the basic decomposition algorithm, we get Algorithm 3.
**Algorithm 3** Improved decomposition algorithm with Layer-Mapping **Input**: An instance of LPNSPI
**Output**: An optimal interdiction plan $x^{*}$ 1:
$\hat{x}\leftarrow 0;\hat{Z}\leftarrow \emptyset ;\underline{V}\leftarrow -\infty ;\bar{V}\leftarrow \infty$
2:
**while** 
$\bar{V}-\underline{V}>0$ 
**do**
3:  
**if** 
$\hat{x}=0$ 
**then**
4:    $x^{0}\leftarrow \hat{x}$; Calculate $w^{0}$ and $K^{0}$ using (4)-(6);
5:    $\hat{w}\leftarrow w^{0}$;
6:  **else**
7:      Solve **Layer-Mapping($x^{0}$,$\hat{x}$)** for $\hat{w}$ and $\hat{K}$;
8:  **end if**9:  Solve [Sub($\hat{w}$)-LM] for $\hat{y}$ and the objective value $V_{\hat{w}}$
10:  $\hat{z}={\hat{K}}^{T}\hat{y}$; $\hat{Z}\leftarrow \hat{Z}\cup \hat{z}$;
11:  
**if** 
$\underline{V}<V_{\hat{w}}$ 
**then**
12:    $x^{\prime }\leftarrow \hat{x}$; $\underline{V}\leftarrow V_{\hat{w}}$;
13:  **end if**
14:  
**if** 
$\bar{V}-\underline{V}\leq 0$ 
**then**
15:    **break**;
16:  **end if**
17:  **if** [Master($\hat{Z}$)-SVI] is feasible **then**
18:    Solve [Master($\hat{Z}$)-SVI] for $\hat{x}$ and the objective value $V_{\hat{Z}}$;
19:    $\bar{V}\leftarrow V_{\hat{Z}}$;
20:  **else**
21:    **break**;
22:  **end if**
23: **end while**
24:
$x^{*}\leftarrow x^{\prime }$
25:
**return** 
$x^{*}$



## 5. A Random-Search Method for Accelerating Convergence

**Figure 2:**
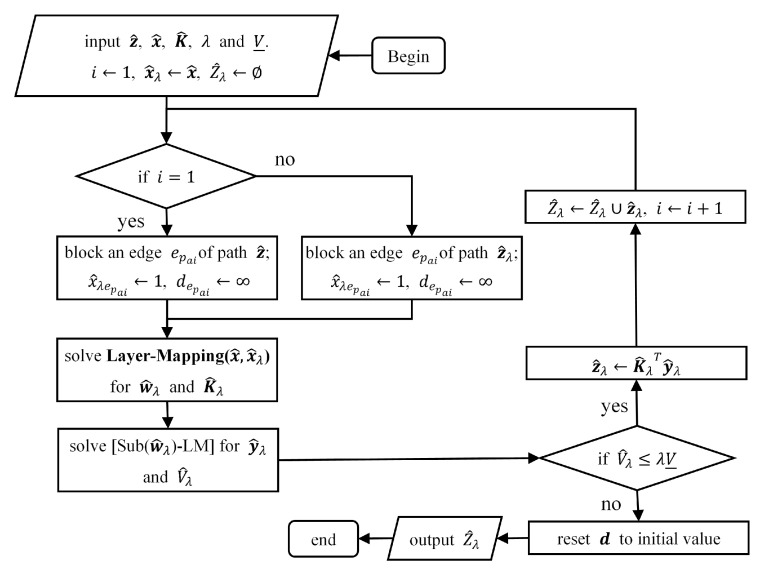
The process of Random-Search.

To accelerate the convergence speed of the decomposition algorithm, we try to use the information of $\hat{z}$ obtained by the subproblem as much as possible in each iteration to limit the feasible domain of the master problem. We propose a Random-Search procedure to increase the number of paths added to $\hat{Z}$ for each iteration, which shares the basic idea of Local-Search proposed by Wood [33] in the NIP of a single-layer network. We hope to find more near-optimal paths in one iteration. However, the existence of inter-layer relationships in multi-layer networks makes the search for near-optimal paths complicated, and the time cost of finding all near-optimal paths in each iteration, which is what Local-Search does, is not small in the face of the large-scale layered network. The process of Random-Search is described as follows. The total communication consumption of these paths is limited by a set constant λ and the current lower bound $\underline{V}$. Let ${\hat{z}}_{\lambda }$ denote an near-optimal path found by Random-Search and let ${\hat{V}}_{\lambda }$ denote the communication cost of this path with an interdiction plan ${\hat{x}}_{\lambda }$ , of which the initial value is set to $\hat{x}$. For a path $\hat{z}$ found by the subproblem, Random-Search first selects one of its edges (denoted as $e_{p_{a1}}$) at random and interdict it, i.e., set ${\hat{x}}_{\lambda_{e_{p_{a1}}}}=1$ and provisionally set $d_{e_{p_{a1}}}=\infty$. Then, for each edge $e_{l}$ in logical layer, recalculate the weight ${\hat{w}}_{e_{l}}$ of it if ${\hat{k}}_{e_{l}e_{p_{a1}}}={\hat{K}}_{e_{l},e_{p_{a1}}}=1$. As for the edges of the logical layer that satisfy ${\hat{K}}_{e_{l},e_{p_{a1}}}=0$, their weights will not change. $e_{p_{a1}}$ does not appear in the physical paths they correspond, so the blocking of $e_{p_{a1}}$ will not change the mapping relationship between them and these paths. Please note that the process of recalculating ${\hat{w}}_{e_{l}}$ can be represented by Layer-Mapping($\hat{x}$,${\hat{x}}_{\lambda }$). The shortest path calculated based on the updated edge weights is a near-optimal path. If the total weight of the path newly found is no more than $\lambda \underline{V}$ , the path will be added into $\hat{Z}$ as ${\hat{z}}_{\lambda }$. After that, Random-Search will choose a new edge $e_{p_{a2}}$ of the path ${\hat{z}}_{\lambda }$ to interdict and repeat the searching process. The process ends when the blocking of edge $e_{p_{an}}$ leads ${\hat{V}}_{\lambda }>\lambda \underline{V}$. Figure 2 shows the execution of Random-Search.

As ${\hat{z}}_{\lambda }\in Z$ is naturally established, adding constraint $V\leq c^{T}{\hat{z}}_{\lambda }+x^{T}D{\hat{z}}_{\lambda }$ to the master problem will not eliminate any optimal solution. We can still use [Master($\hat{Z}$)-SVI] with the $\hat{Z}$ extended by Random-Search even if (22) and (23) are not supervalid (for convenience, we name these inequalities “λ-SVIs”). Since we keep ${\hat{V}}_{\lambda }>\lambda \underline{V}$ in every iteration, adding corresponding λ-SVIs to [Master($\hat{Z}$)-SVI] will finally lead to an approximate objective value $V_{\lambda }^{*}$ which satisfies $V_{\lambda }^{*}\geq \frac{1}{\lambda }V^{*}$. We give the properties of λ-SVIs and prove it as follows.

**Theorem** **1.** For an interdiction plan $\hat{x}$ given by [Master($\hat{Z}$)] during an iteration of Algorithm 2, let ${\hat{z}}_{\lambda }$ denote a feasible solution of [Sub($\hat{x}$)] that may be non-optimal. Let ${\hat{V}}_{\lambda }=\sum_{e_{p}\in A_{p}}\left(c_{e_{p}}+{\hat{x}}_{e_{p}}d_{e_{p}}\right){\hat{z}}_{\lambda_{e_{p}}}$ and let $V^{*}$ denote the global optimal objective value of [Master(Z)]. Then Type-I inequality ${\hat{z}}_{\lambda }^{T}x\geq 1$ does not eliminate all optimal solutions of [Master(Z)] unless the incumbent solution $\hat{x}$ leads to a lower bound $\underline{V}\geq \frac{1}{\lambda }V^{*}$, providing that ${\hat{V}}_{\lambda }\leq \lambda \underline{V}$.

**Proof of Theorem** **1.** Let $x^{*}$ denote the global optimal solution of the defender’s interdiction plan. Assuming that $\underline{V}<\frac{1}{\lambda }V^{*}$ during an iteration, if Type-I inequality ${\hat{z}}_{\lambda }^{T}x\geq 1$ eliminates all optimal solutions, which means ${\hat{z}}_{\lambda }^{T}x^{*}=0$, then

$$\begin{array}{cc} V^{*} & \leq c^{T}{\hat{z}}_{\lambda }+x^{{*}^{T}}D{\hat{z}}_{\lambda }\;\;\;(\mathrm{is}\;\mathrm{naturally}\;\mathrm{established})\\ \; & =c^{T}{\hat{z}}_{\lambda }\;\;(\mathrm{because}\;{\hat{z}}_{\lambda }^{T}x^{*}=0)\\ \; & ={\hat{V}}_{\lambda }-{\hat{x}}^{T}D{\hat{z}}_{\lambda }\\ \; & \leq {\hat{V}}_{\lambda }\\ \; & \leq \lambda \underline{V}\\ \; & <V^{*}\;\;(\mathrm{which}\;\mathrm{is}\;a\;\mathrm{contradiction}) \end{array}$$

□

In fact, the two types of SVIs and their corollaries can be modified to apply to a non-optimal pathfinding result $\hat{z}$ if $\hat{z}$ is an approximate optimal solution of the current subproblem [Sub($\hat{x}$)]. The modified inequalities are not supervalid, which means that the inequalities may eliminate all optimal solutions. However, when this happens, we will already have an approximate global optimal interdiction solution.

**Corollary** **1.** *For a feasible solution ${\hat{z}}_{\lambda }$ of [Sub($\hat{x}$)], which leads to an objective value ${\hat{V}}_{\lambda }\leq \lambda \underline{V}$. Order the $d_{m}{\hat{z}}_{\lambda_{m}}>0$ so that $d_{m_{1}}{\hat{z}}_{\lambda m_{1}}\geq d_{m_{2}}{\hat{z}}_{\lambda m_{2}}\ldots \geq d_{m_{H}}{\hat{z}}_{\lambda m_{H}}$. Then, if $\lambda \underline{V}\geq c^{T}{\hat{z}}_{\lambda }+\sum_{h=1}^{p}d_{m_{h}}{\hat{z}}_{\lambda m_{h}}$ for p≤H, the Type-I inequality of Theorem 1 can be tightened to ${\hat{z}}_{\lambda }^{T}x\geq p+1$*

**Proof of Corollary** **1.** Assuming that ${\hat{z}}_{\lambda }^{T}x\leq p$, then

$$\begin{array}{cc} V^{*} & \leq c^{T}{\hat{z}}_{\lambda }+x^{{*}^{T}}D{\hat{z}}_{\lambda }\\ \; & \leq c^{T}{\hat{z}}_{\lambda }+\sum_{h=1}^{p}d_{m_{h}}{\hat{z}}_{\lambda_{m_{h}}}\;\;(\mathrm{because}\;{\hat{z}}_{\lambda }^{T}x\leq p\;\mathrm{and}\;{\hat{z}}_{\lambda_{m_{h}}}\in N\;\mathrm{imply}\;\mathrm{that}\\ & \;\;\;\;\mathrm{the}\;\mathrm{number}\;\mathrm{of}\;\mathrm{edges}\;\mathrm{selected}\;\mathrm{by}\;\mathrm{both}\;{\hat{z}}_{\lambda }\;\mathrm{and}\;x^{*}\;\mathrm{is}\;\mathrm{no}\;\mathrm{more}\;\mathrm{than}\;p)\\ \; & \leq \lambda \underline{V}<V^{*}\;\;(\mathrm{which}\;\mathrm{is}\;a\;\mathrm{contradiction}) \end{array}$$

□

**Theorem** **2.** For an interdiction plan $\hat{x}$ given by [Master($\hat{Z}$)] during an iteration of Algorithm 2, let ${\hat{z}}_{\lambda }$ denote a feasible solution of [Sub($\hat{x}$)] that may be non-optimal. Let ${\tilde{z}}_{\lambda }=\left(diag\left(1-\hat{x}\right)\right){\hat{z}}_{\lambda }$. Then, the Type-II inequality ${\tilde{z}}_{\lambda }^{T}x\geq 1$ does not eliminate all optimal solutions of [Master(Z)] unless the incumbent solution $\hat{x}$ leads to a lower bound $\underline{V}\geq \frac{1}{\lambda }V^{*}$, providing that ${\hat{V}}_{\lambda }\leq \lambda \underline{V}$.

**Proof of Theorem** **2.** Assuming that $\underline{V}<\frac{1}{\lambda }V^{*}$, if Type-II inequality ${\tilde{z}}_{\lambda }^{T}x\geq 1$ eliminates all optimal solutions, which means ${\tilde{z}}_{\lambda }^{T}x^{*}=x^{{*}^{T}}\left(diag\left(1-\hat{x}\right)\right){\hat{z}}_{\lambda }=x^{{*}^{T}}\left(I-diag\left(\hat{x}\right)\right){\hat{z}}_{\lambda }=0$, we can obtain that $x^{{*}^{T}}\left(I-diag\left(\hat{x}\right)\right)D{\hat{z}}_{\lambda }=0$ because all elements in vector $x^{{*}^{T}}\left(I-diag\left(\hat{x}\right)\right)$ are 0 or 1. Then

$$\begin{array}{cc} V(x^{*},{\hat{z}}_{\lambda }) & =c^{T}{\hat{z}}_{\lambda }+x^{{*}^{T}}D{\hat{z}}_{\lambda }\\ \; & =c^{T}{\hat{z}}_{\lambda }+x^{{*}^{T}}\left(diag\left(\hat{x}\right)\right)D{\hat{z}}_{\lambda }\\ \; & \leq c^{T}{\hat{z}}_{\lambda }+\hat{x}D{\hat{z}}_{\lambda }={\hat{V}}_{\lambda }\\ \; & \leq \lambda \underline{V}\\ \; & <V^{*}\;\;(\mathrm{which}\;\mathrm{contradicts}\;\mathrm{the}\;\mathrm{fact}\;\mathrm{that}\\ \; & \;\;\;\;x^{*}\;\mathrm{and}\;V^{*}\;\mathrm{are}\;\mathrm{global}\;\mathrm{optimal}\;\mathrm{solutions}) \end{array}$$

□

**Corollary** **2.** *For a Type-II inequality ${\tilde{z}}_{\lambda_{1}}^{T}x\geq 1$, let ${\tilde{M}}_{\lambda_{1}}=\left\{m|{\tilde{z}}_{\lambda_{1m}}=1\right\}$ and let ${\tilde{M}}_{\lambda_{2}}$ be any subset of ${\tilde{M}}_{\lambda_{1}}$ such that $\sum_{m\in {\tilde{M}}_{\lambda_{2}}}d_{m}+\left(c^{T}{\hat{z}}_{\lambda_{1}}+{\hat{x}}_{1}^{T}D{\hat{z}}_{\lambda_{1}}\right)\leq \lambda \underline{V}$. Then, the Type-II SVI of Theorem 2 can be tightened to ${\tilde{z}}_{\lambda_{2}}^{T}x\geq 1$ , where ${\tilde{z}}_{\lambda_{2m}}={\tilde{z}}_{\lambda_{1m}}$ for all $m\in {\tilde{M}}_{\lambda_{1}}\backslash {\tilde{M}}_{\lambda_{2}}$ and 0 otherwise.*

**Proof of Corollary** **2.** Assuming that $\underline{V}<\frac{1}{\lambda }V^{*}$, if Type-II inequality ${\tilde{z}}_{\lambda_{2}}^{T}x\geq 1$ eliminates all optimal solutions, which means ${\tilde{z}}_{\lambda_{2}}^{T}x^{*}=0$, let ${\hat{M}}_{\lambda_{1}}=\left\{m|{\hat{z}}_{\lambda_{1m}}\geq 1\right\}$, then

$$\begin{array}{cc} V(x^{*},{\hat{z}}_{\lambda_{1}}) & =c^{T}{\hat{z}}_{\lambda }+x^{{*}^{T}}D{\hat{z}}_{\lambda }\\ \; & =\sum_{m\in ({\hat{M}}_{\lambda_{1}}\backslash {\tilde{M}}_{\lambda_{1}})}\left(c_{m}{\hat{z}}_{\lambda_{m}}+x_{m}^{*}d_{m}{\hat{z}}_{\lambda_{m}}\right)+\sum_{m\in {\tilde{M}}_{\lambda_{2}}}\left(c_{m}{\hat{z}}_{\lambda_{m}}+x_{m}^{*}d_{m}{\hat{z}}_{\lambda_{m}}\right)+\\ \; & \;\;\;\;\sum_{m\in ({\tilde{M}}_{\lambda_{1}}\backslash {\tilde{M}}_{\lambda_{2}})}\left(c_{m}{\hat{z}}_{\lambda_{m}}+x_{m}^{*}d_{m}{\hat{z}}_{\lambda_{m}}\right)\\ \; & =\sum_{m\in ({\hat{M}}_{\lambda_{1}}\backslash {\tilde{M}}_{\lambda_{1}})}\left(c_{m}{\hat{z}}_{\lambda_{m}}+x_{m}^{*}d_{m}{\hat{z}}_{\lambda_{m}}\right)+\sum_{m\in {\tilde{M}}_{\lambda_{2}}}\left(c_{m}{\hat{z}}_{\lambda_{m}}+x_{m}^{*}d_{m}{\hat{z}}_{\lambda_{m}}\right)\\ \; & =\sum_{m\in {\tilde{M}}_{\lambda_{2}}}x_{m}^{*}d_{m}{\hat{z}}_{\lambda_{m}}+\left[\sum_{m\in ({\hat{M}}_{\lambda_{1}}\backslash {\tilde{M}}_{\lambda_{1}})}\left(c_{m}{\hat{z}}_{\lambda_{m}}+x_{m}^{*}d_{m}{\hat{z}}_{\lambda_{m}}\right)+\sum_{m\in {\tilde{M}}_{\lambda_{2}}}c_{m}{\hat{z}}_{\lambda_{m}}\right]\\ \; & \leq \sum_{m\in {\tilde{M}}_{\lambda_{2}}}d_{m}+\left(c^{T}{\hat{z}}_{\lambda_{1}}+{\hat{x}}_{1}^{T}D{\hat{z}}_{\lambda_{1}}\right)\\ \; & \leq \lambda \underline{V}<V^{*}\;\;(\mathrm{which}\;\mathrm{leads}\;\mathrm{to}\;a\;\mathrm{contradiction}) \end{array}$$

□

Based on Algorithm 3, we add the Random-Search module and get Algorithm 4 as follows:
**Algorithm 4** Improved decomposition algorithm with Layer-Mapping and Random-Search **Input**: An instance of LPNSPI and a tolerable approximation ratio $\frac{1}{\lambda }$**Output**: A tolerable near-optimal interdiction plan $x_{\lambda }^{*}$ 1:
$\hat{x}\leftarrow 0;\hat{Z}\leftarrow \emptyset ;\underline{V}\leftarrow -\infty ;\bar{V}\leftarrow \infty$
2:
**while** 
$\bar{V}-\underline{V}>0$ 
**do**
3:  
**if** 
$\hat{x}=0$ 
**then**
4:    $x^{0}\leftarrow \hat{x}$; Calculate $w^{0}$ and $K^{0}$ using (4)-(6);
5:    $\hat{w}\leftarrow w^{0}$;
6:  **else**
7:    Solve **Layer-Mapping($x^{0}$,$\hat{x}$)** for $\hat{w}$ and $\hat{K}$;
8:  **end if**9:  Solve [Sub($\hat{w}$)-LM] for $\hat{y}$ and the objective value $V_{\hat{w}}$;
10:  $\hat{z}={\hat{K}}^{T}\hat{y}$; $\hat{Z}\leftarrow \hat{Z}\cup \hat{z}$;
11:  
**if** 
$\underline{V}<V_{\hat{w}}$ 
**then**
12:    $x^{\prime }\leftarrow \hat{x}$; $\underline{V}\leftarrow V_{\hat{w}}$;
13:  **end if**
14:  
**if** 
$\bar{V}-\underline{V}\leq 0$ 
**then**
15:    **break**;
16:  **end if**
17:  Solve Random-Search($\hat{x},\hat{z},\hat{K},\lambda ,\underline{V}$) for ${\hat{Z}}_{\lambda }$ ;
18:  $\hat{Z}\leftarrow \hat{Z}\cup {\hat{Z}}_{\lambda }$;
19:  **if** [Master($\hat{Z}$)-SVI] is feasible **then**
20:    Solve [Master($\hat{Z}$)-SVI] for $\hat{x}$ and the objective value $V_{\hat{Z}}$;
21:    $\bar{V}\leftarrow V_{\hat{Z}}$;
22:  **else**
23:    **break**;
24:  
**end if**
25: **end while**
26:
$x_{\lambda }^{*}\leftarrow x^{\prime }$
27:
**return** 
$x_{\lambda }^{*}$



## 6. Computational Experiments

We tested our algorithms in a set of generated layered networks with directed random networks as their logical layers. Random networks, small-world networks, scale-free networks and grid networks are used as physical layer in these instances. The network in the logical layer are smaller than the physical-layer network in each instance, and each node of the logical layer correspond a randomly selected node of the physical layer, which means that these two nodes share the same entity. We generated random networks by connecting newly added nodes to previous nodes with a certain probability *p*. *p* is adjusted for each instance to ensure that all the random networks have the same expected average degree. The small-world networks are generated by first constructing a nearest-neighbor coupling network and then reconnecting the edges with different probabilities. For the scale-free case, we first generate a small random network, and then assign the connection probabilities according to the degrees of nodes, and finally preferentially connect the newly added nodes to the nodes with better connectivity in the light of the probabilities. As for the grid networks, conventional square lattice networks are used. The communication costs of edges $c_{e_{p}}$s and the interdiction increments $d_{e_{p}}$s in physical layer are integers that are randomly distributed on [1, 20] and [200, 1000], respectively. The resource consumption of interdiction r is set to 1 in practice. A time limit of 3600 s is set for each experiment.

Table 2 shows the parameters of the test problems we used. The blank cells repeat values from cells above. The numbers in the brackets of the column “$N_{l}$” and column “$N_{p}$” represent the average degree of the generated networks. For each problem we generated ten instances. We programmed the algorithms presented above using the MATLAB toolbox YALMIP and CPLEX 12.8 callable library. Computation is performed on a Windows 10 64-bit laptop with an Intel Core i7-9700K CPU (3.60 GHZ) and 16 GB of RAM.

The value of λ in Algorithm 4 is set as 1.05, 1.10 and 1.15, respectively, and the error range of the corresponding optimal objective value is 95.2%, 90.9% and 87.0%. The basic results for LPNSPI are shown in Table 3. The column “instance” represents the number of instances we used for comparison in the problem of a certain scale. *T*, average solution time in seconds; Std.T, the standard deviation of the solution time; *N*, average iteration times; Std.N, the standard deviation of the iteration times. The numbers in brackets of the column “*T*” indicate the number of the instances which were solved within 3600 s. The “–”s of the same rows indicates “not applicable” because there was at least one instance which was not successfully solved within the allotted time. It can be intuitively seen from Table 3 that the Layer-Mapping module make Algorithm 3 much faster than the basic decomposition algorithm with almost the same number of iterations, especially when the scale of the physical layer is large. However, despite this, Algorithm 3 cannot solve all the instances within the stipulated time. Combining Random-Search with Layer-Mapping, Algorithm 3 takes significantly less time and fewer iterations, and successfully solved all the instances. It should be noted that among the ten instances of ’gn1000’, there are two extreme instances, which increase the average solving time of ’gn1000’ problem (even more than the average solving time of ’gn2000’). However, it does not affect the comparison between the performance of different algorithms.

To analyze the specific running time of each part (i.e., the master problem, the subproblem and the Random-Search part) of the three algorithms, we selected some “easy” instances of the hardest problems of each network type in Table 3 (that is, rd20000, sf20000, sw20000 , gn1000 and gn2000), where “easy" means that all three algorithms can solve the problem within 3600 s. The results are shown in Table 4. T.M is the running time of the master problem; T.S is the running time of the subproblem; T.RS is the time spent on Random-Search. Initialization, formatting, and other parts of the program only account for a small portion of the time and are not listed. Err is the average error rate of the results obtained by Algorithm 4, and the numbers in parentheses indicate the number of instances where Algorithm 3 find a near-optimal solution rather than an exact optimal solution. Std.Err is the standard deviation of Err. As Table 4 shows, Algorithm 3 takes almost the same time in solving the master problem as Algorithm 1, but takes much less time to solve the subproblem. With Random-Search, Algorithm 3 greatly reduces the time of the master problem and the subproblem, but it also takes a considerable amount of time in Random-Search to find near-optimal paths.

A suitable value of λ allows Random-Search to find a large number of near-optimal paths, but at the same time, the final result may have some errors from the actual optimal solution. In our test instances, Algorithm 3 solved many instances accurately, with an overall average accuracy of more than 99.7%. Of course, we can set the value of λ to 0, which will make the final result the global optimal solution, but also make it difficult for Random-Search to find near-optimal paths. We tested Algorithm 3 on the case when λ=0, and found that the running time results are not so satisfying because much fewer near-optimal paths were found.

To study the effect of the value of λ on the solution speed of Algorithm 3, we set three cases of λ=1.05, λ=1.1, and λ=1.15, and tested the algorithm on all the instances of the problems in Table 4. Table 5 compares the algorithm time, number of iterations, and errors of the near-optimal solution in the three cases. We used all ten instances of each problem when comparing the running time and the number of iterations of the algorithm. However, for “rd20000”, “sf20000” and “sw20000”, there were instances where Algorithm 1 and Algorithm 2 could not give the exact optimal results. The numbers in parentheses in the column “Err” indicate the number of instances used in calculating the average error rate and the number of non-optimal solutions given by Algorithm 3. For example, “0.25%(2 in 8)” means that in 8 verifiable instances, Algorithm 3 gives 2 approximate solutions (and 6 exact solutions), with an average error of 0.25%.

Due to the randomness of the pathfinding results of Random-Search, the number of near-optimal paths found in a specific iteration will fluctuate, and the contribution of these paths and the corresponding SVIs to the master problem is also uncertain. In some instances, the algorithm took more time with a larger λ than with a smaller one. However, in general, as Table 5 shows, a slightly larger λ makes the algorithm converge faster.

**Figure 3:**
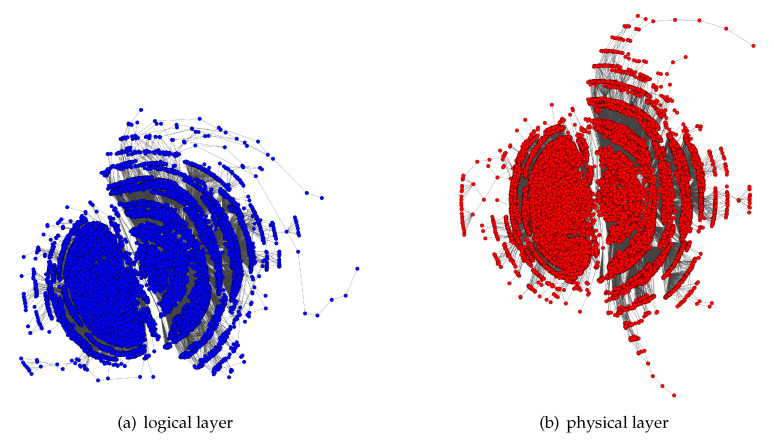
The structure of a real bi-layer IoT network (after data cleaning).

Furthermore, we examine our improving methods in commercial data of a bi-layer network which is obtained from scanning over the Internet. This bi-layer network includes a physical layer of 36,409 nodes and 49,084 edges, and a logical layer of 32,490 nodes and 51,340 edges. The physical nodes contain switching equipment, terminal equipment, storage equipment, control equipment, etc., and they are connected by physical links which consist of optical fibers, twisted-pair lines, coaxial cables, wireless media links, etc. On the logical layer, nodes represent operating systems and other software, and links represent information transfers, remote logins, network sessions, etc. The network structure is shown in Figure 3.

After data cleaning of the original network (remove isolated nodes and connected components which do not include the source-destination pair), we designed experiments to test the changes of the algorithm running time and blocking effect with the increase of interdiction resources. The time limit is set to 3600 s.

**Figure 4:**
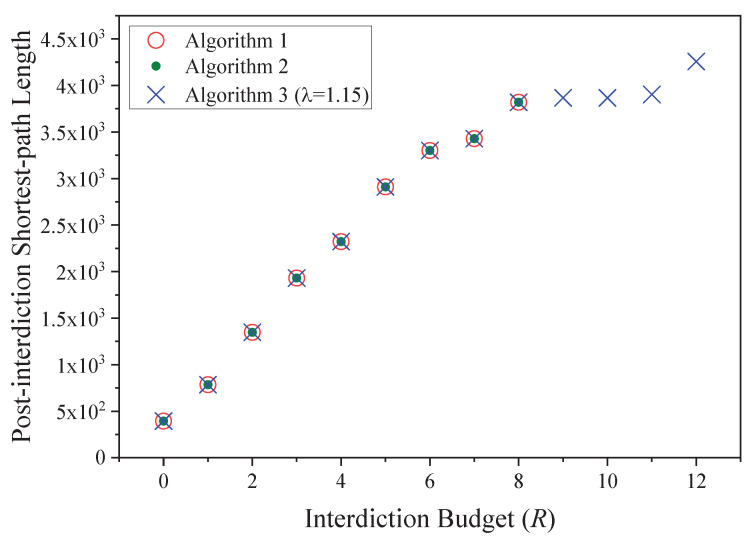
Interdiction effects in the bi-layer IoT network.

As shown in Table 6, the processing time of the algorithms increases rapidly when the number of interdicted edges (*R*) grows larger. Both Algorithm 3 and Algorithm 4 solve the problem with much less time than Algorithm 1 (benchmark) when interdicted edges are no more than 8, which confirms the effectiveness of Layer-Mapping. As a large number of near-optimal paths are found by Random-Search, when *R* is small, the solution of Algorithm 3 is faster than that of Algorithm 4. However, when *R* grows up to 9, neither Algorithm 1 nor Algorithm 3 can solve the problem within 3600 s. Algorithm 3 with both Layer-Mapping and Random-Search successfully solves the problem when *R* is no more than 12. The interdiction effects of the three algorithms are compared in Figure 4. The objective function, which is the length of the shortest $s_{l}$-$t_{l}$ path, increases when *R* grows. We find that even if the λ in Algorithm 4 is set to 1.15, which leads to a potential error range of 87%, accurate optimal solutions are found in comparable cases (i.e., when *R* is no more than 8).

## 7. Conclusions

This paper focuses on blocking malicious network behaviors in IoT systems which can be modeled as logical–physical networks. The problem is represented as the shortest-path interdiction problem in layered networks, where the target paths and the interdiction behaviors are on different layers of the network. The attacker seeks to minimize the total communication cost of the attacking path from the source node to the target node on the logical layer, and the defender aims to maximize this path by interdicting edges on the physical layer. The interdiction of edges on the physical layer affects edges on the logical layer through the inter-layer relationship. In this problem, every node in the logical-layer network has a corresponding node on the physical layer, and the weight of each logical-layer edge is decided by a shortest path on the physical layer, with the endpoints of the logical-layer edge being the start node and the end node.

By referring to the experience of interdiction problems in monolayer networks, we model LPNSPI as a solvable form of Benders decomposition algorithm and apply “supervalid inequalities” (SVIs) on it. A Layer-Mapping module is proposed to deal with the explicit non-linearity of the objective function and reduce double counting of the subproblem. Layer-Mapping recalculates the status of the physical layer, basing on the current solution of the master problem and the initial network status. To accelerate the convergence of the decomposition algorithm, we raise Random-Search. By specifying an acceptable approximation range, Random-Search can randomly find multiple near-optimal paths in an iteration; as a result these paths and the corresponding SVIs can be added as constraints to the master problem. Computational results show the effectiveness of Layer-Mapping and Random-Search.

## Figures and Tables

**Table 1:** Comparisons with LPNSPI and other layered network interdiction problems.

	Kennedy [30]	Wei et al. [31]	Baycik et al. [32]	LPNSPI
objective function	Minimize the maximum flow	Maximizing the shortest path	Minimize the maximum flow	Maximizing the shortest path
inter-layer relationship	geographical co-located relations	logic constraints	flow requirement constraints	path constraints
duality	Yes	Yes	Yes	No

**Table 2:** Test problem statistics.

Problem Name	Type of $G_{p}$	Type of $G_{l}$	$N_{l}$	$N_{p}$	*R*
rd2000	random	random	100 (3)	2000 (2)	2
rd5000			200 (3)	5000 (2)	3
rd10000			500 (3)	10,000 (2)	4
rd20000			1000 (3)	20,000 (2)	5
sf2000	scale-free		100(3)	2000 (3)	2
sf5000			200 (3)	5000(3)	3
sf10000			500 (3)	10,000 (3)	4
sf20000			1000 (3)	20,000 (3)	5
sw2000	small-world		100 (3)	2000 (4)	2
sw5000			200 (3)	5000 (4)	3
sw10000			500 (3)	10,000 (4)	4
sw20000			1000 (3)	20,000 (4)	5
gd1000	grid		100 (3)	1000 (2)	2
gd2000			100 (3)	2000 (2)	2

**Table 3:** The computational results for networks of different scale and different types.

Problem	Algorithm 1				Algorithm 3				Algorithm 4 (λ=1.05)			
	*T*	*Std.T*	*N*	*Std.N*	*T*	*Std.T*	*N*	*Std.N*	*T*	*Std.T*	*N*	*Std.N*
rd2000	5.7	2.5	17.3	6.9	3.5	1.4	17.3	6.9	3.0	1.2	8.9	4.8
rd5000	22.4	12.9	30.4	16.5	8.7	5.8	30.4	16.5	6.9	3.2	11.7	5.7
rd10000	163.5	113.7	64.3	43.6	39.2	33.5	64.3	43.6	16.5	9.3	14.3	10.6
rd20000	(7)	–	–	–	(8)	–	–	–	86.5	132.8	25.0	26.7
sf2000	8.4	2.7	24.1	6.8	5.3	1.8	24.1	6.8	3.4	1.2	11.1	3.2
sf5000	39.9	20.3	47.1	22.5	17.8	11.4	47.1	22.5	8.4	3.3	15.6	5.5
sf10000	406.2	272.4	127.2	75.1	134.6	118.9	127.2	75.1	28.5	8.9	23.3	6.7
sf20000	(8)	–	–	–	(9)	–	–	–	95.4	39.9	26.4	7.2
sw2000	7.2	2.9	19.7	6.6	4.4	1.6	19.7	6.6	2.6	0.6	8.7	2.3
sw5000	46.1	30.9	49.1	28.0	20.2	15.7	49.1	28.0	6.3	2.0	12.8	4.0
sw10000	560.2	518.8	145.2	111.2	224.2	264.6	145.2	111.2	26.6	14.8	23.9	12.4
sw20000	(6)	–	–	–	(7)	–	–	–	84.0	41.3	27.7	12.7
gn1000	933.2	969.7	579.6	359.8	812.7	867.4	579.6	359.8	192.0	144.8	57.1	31.0
gn2000	757.3	675.9	488.4	267.8	655.5	622.1	488.4	276.8	157.5	126.7	45.0	24.9

**Table 4:** The running time of the main parts and the error of Algorithm 4.

Problem	Instance	Algorithm 1		Algorithm 3		Algorithm 4 (λ=1.05)				
		*T.M*	*T.S*	*T.M*	*T.S*	*T.M*	*T.S*	*T.RS*	*Err*	*Std.Err*
rd20000	7	108.7	695.0	107.2	16.9	6.9	9.3	13.6	0.28% (2)	0.005
sf20000	8	300.9	1435.0	313.4	67.4	19.5	14.8	48.9	0.11% (1)	0.003
sw20000	6	321.0	1473.0	337.1	29.4	20.7	12.3	29.0	0 (0)	0
gn1000	10	746.7	183.5	740.6	69.0	97.9	6.5	87.2	0.23% (5)	0.003
gn2000	10	582.7	171.2	597.8	54.2	91.2	4.4	61.4	0.30% (4)	0.005

**Table 5:** Results for Algorithm 4 when λ changes.

Problem	Algorithm 4								
	λ=1.05			λ=1.1			λ=1.15		
	***T***	***N***	***Err***	***T***	***N***	***Err***	***T***	***N***	***Err***
rd20000	86.5	25.0	0.25% (2 in 8)	48.9	14.3	0.15% (1 in 8)	36.4	9.7	0.46% (2 in 8)
sf20000	95.4	26.4	0.10% (1 in 9)	83.3	19.7	0.66% (4 in 9)	75.1	15.4	1.91% (5 in 9)
sw20000	84.0	27.7	0 (0 in 7)	66.2	18.5	0 (0 in 7)	51.0	11.7	0.50% (1 in 7)
gn1000	192.0	57.1	0.23% (5 in 10)	110.1	29.6	0.69% (7 in 10)	75.6	17.0	1.17% (8 in 10)
gn2000	157.5	45.0	0.30% (4 in 10)	89.9	25.1	0.68% (6 in 10)	74.0	16.7	0.81% (8 in 10)

**Table 6:** The processing time when defender’s resources increase.

R	Processing Time		
	Algorithm 1	Algorithm 3	Algorithm 4 (λ=1.15)
1	81.3	18.7	48.4
2	187.8	33.5	55.5
3	275.5	51.6	74.5
4	299.0	92.4	103.5
5	258.2	81.8	116.9
6	265.0	115.8	83.6
7	605.2	276.4	211.6
8	828.2	403.0	292.9
9	-	-	384.2
10	-	-	499.5
11	-	-	1528.7
12	-	-	2456.1
